# Implementing Patient-Derived Xenografts to Assess the Effectiveness of Cyclin-Dependent Kinase Inhibitors in Glioblastoma

**DOI:** 10.3390/cancers11122005

**Published:** 2019-12-12

**Authors:** Janis J. Noonan, Monika Jarzabek, Frank A. Lincoln, Brenton L. Cavanagh, Arhona R. Pariag, Viktorija Juric, Leonie S. Young, Keith L. Ligon, Hanne Jahns, Daniella Zheleva, Jochen H. M. Prehn, Markus Rehm, Annette T. Byrne, Brona M. Murphy

**Affiliations:** 1 Department of Physiology & Medical Physics, Royal College of Surgeons in Ireland, D02 YN77 Dublin 2, Ireland; janisjnoonan@gmail.com (J.J.N.); monikajarzabek@rcsi.ie (M.J.); franklincoln@rcsi.ie (F.A.L.); arhonapariag@rcsi.ie (A.R.P.); viktorijajuric@rcsi.ie (V.J.); jprehn@rcsi.ie (J.H.M.P.); annettebyrne@rcsi.ie (A.T.B.); 2 Cellular and Molecular Imaging Core, Royal College of Surgeons in Ireland, D02 YN77 Dublin 2, Ireland; brentoncavanagh@rcsi.ie; 3 Endocrine Oncology Research Group, Department of Surgery, Royal College of Surgeons in Ireland, D02 YN77 Dublin 2, Ireland; lyoung@rcsi.ie; 4 Department of Oncologic Pathology, Dana-Farber Cancer Institute, 450 Brookline Avenue, Boston, MA 02215, USA; keith_ligon@dfci.harvard.edu; 5 Pathobiology Section, School of Veterinary Medicine, University College Dublin, D02 YN77 Dublin 4, Ireland; hanne.jahns@ucd.ie; 6 Cyclacel Ltd., 1 James Lindsay Place, Dundee, Scotland DD1 5JJ, UK; dzheleva@cyclacel.com; 7 Institute of Cell Biology and Immunology, University of Stuttgart, D-70569 Stuttgart, Germany; markus.morrison@izi.uni-stuttgart.de; 8 Stuttgart Research Center Systems Biology, University of Stuttgart, D-70569 Stuttgart, Germany

**Keywords:** glioblastoma, CDK inhibitors, seliciclib, CYC065, TRAIL, drozitumab, neurospheres, patient-derived xenograft

## Abstract

Glioblastoma (GBM) is the most common primary brain tumor with no available cure. As previously described, seliciclib, a first-generation cyclin-dependent kinase (CDK) inhibitor, down-regulates the anti-apoptotic protein, Mcl-1, in GBM, thereby sensitizing GBM cells to the apoptosis-inducing effects of the death receptor ligand, tumor necrosis factor-related apoptosis-inducing ligand (TRAIL). Here, we have assessed the efficacy of seliciclib when delivered in combination with the antibody against human death receptor 5, drozitumab, in clinically relevant patient-derived xenograft (PDX) models of GBM. A reduction in viability and significant levels of apoptosis were observed in vitro in human GBM neurospheres following treatment with seliciclib plus drozitumab. While the co-treatment strategy induced a similar effect in PDX models, the dosing regimen required to observe seliciclib-targeted responses in the brain, resulted in lethal toxicity in 45% of animals. Additional studies showed that the second-generation CDK inhibitor, CYC065, with improved potency in comparison to seliciclib, induced a significant decrease in the size of human GBM neurospheres in vitro and was well tolerated in vivo, upon administration at clinically relevant doses. This study highlights the continued need for robust pre-clinical assessment of promising treatment approaches using clinically relevant models.

## 1. Introduction

Glioblastoma (GBM) is the most common and aggressive primary brain tumor that occurs in humans. Despite intense effort to combat GBM with surgery, radiation and genotoxic chemotherapy, 90–95% of patients typically succumb to the disease within 5 years of diagnosis [1,2], with nearly all patients experiencing disease relapse, usually within 6–8 months of treatment onset [3]. Given this poor prognosis, it is critical that new and better treatments are identified. 

There are a number of factors that contribute to the limited effectiveness of current therapies, which need to be overcome for future treatment strategies. One of the most critical factors is the extreme resistance of GBM to apoptosis-inducing stimuli [4,5]. Apoptotic cell death is primarily achieved through the cleavage of numerous cellular proteins by the proteolytic caspase enzymes [6,7,8,9]. Two major pathways lead to caspase activation in mammalian cells: the extrinsic or death receptor pathway and the intrinsic mitochondrial pathway [6,10]. The extrinsic apoptotic pathway can be induced when extracellular ligands bind to death receptors, e.g., ligation of tumor necrosis factor-related apoptosis-inducing ligand (TRAIL) to DR4/TRAIL-R1 and DR5/TRAIL-R2, leading to formation of the death-inducing signaling complex (DISC) and subsequent activation of procaspase-8. Extrinsic apoptotic signaling may then be propagated either via a mitochondrial-independent or a mitochondrial-dependent pathway. 

Temozolomide (TMZ) chemotherapy and radiotherapy, the current standard of care for GBM patients, typically engage the mitochondrial pathway of apoptosis to induce death within their target populations [11,12]. The mitochondrial pathway is primarily induced by changes in Bcl-2 family interactions on the outer mitochondrial membrane, which culminates in pore formation. Mitochondrial outer membrane permeabilization results in the release of cytochrome *c*, subsequent formation of the apoptosome, and activation of procaspase-9, leading in turn to a cascade of caspase-mediated cleavage reactions and ultimately cell death [13]. 

As a result of the overexpression of numerous survival proteins and attenuated levels of several pro-apoptotic proteins [12,14,15], GBM is able to resist the execution of both major caspase activation pathways. Therefore, therapies that aim to modulate these pathways hold promise as potential new treatment options for patients. 

Recent efforts to sensitize GBM to apoptosis have focused on death receptors ligands, such as TRAIL to activate the extrinsic pathway of apoptosis [16]. Numerous studies have demonstrated that several malignancies, including GBM, are resistant to TRAIL monotherapy, due in part to their overexpression of anti-apoptotic proteins, such as Mcl-1 [16,17]. Instead, combination strategies are required to elicit maximum impact [18,19]. We have previously demonstrated that reducing the expression of Mcl-1, is sufficient to sensitize previously resistant GBM cells to TRAIL therapy [20]. To down-regulate Mcl-1 expression, we utilized the cyclin-dependent kinase (CDK)-inhibitor, seliciclib.

CDKs are critical regulatory enzymes that drive all cell cycle transitions [21,22]. CDK-1, -2, -4, and -6 in particular, directly promote cell cycle progression, while CDKs, such as CDK7, -8 and -9 regulate transcription. Other CDKs have more specialized functions. For instance, CDK5 regulates developmental and adult neurogenesis, as well as cell survival in post-mitotic neurons [23]. Due to the central role of CDKs in the control of cell division, it is unsurprising that virtually all cancers, including GBM, harbor genomic alterations that lead to the constitutive activation of CDKs, resulting in the proliferation of cancer cells [24]. Such observations have resulted in the expansion of translational research in the CDK inhibitor space, in particular for those tumors that are resistant to established treatments. 

Inhibitors developed to block cell cycle regulation by CDK4/6 have achieved the greatest clinical results to date. The selective CDK4/6 inhibitors, palbociclib, ribociclib and abemaciclib, are currently in clinical use for the treatment of breast cancer [25,26,27,28]. However, the potential of these and other CDK inhibitors to improve GBM patient survival requires further pre-clinical assessment [29], especially in appropriate in vitro and in vivo models.

The aim of the present study was to evaluate CDK inhibitors, administered both alone and in combination with death receptor ligation, as a potential novel therapeutic approach in GBM. We employed two CDK inhibitors: seliciclib, a first generation compound [30], that primarily targets CDK -2,-5,-7 and -9; and CYC065, its second-generation derivative [31] that is more specific for CDK -2, -5 and -9. To target the TRAIL signaling pathway, we selected a monoclonal antibody specifically directed against human TRAIL-R2 (drozitumab) [32], as GBM cell lines predominately express TRAIL-R2 [33]. We examined the effectiveness of a single and dual treatment strategy in human GBM neurospheres and in an orthotopic patient-derived xenograft (PDX) model. PDX models better recapitulate the physiological characteristics of patient tumors compared with the monolayer systems that we have previously employed [20]. For instance, human GBM neurosphere cultures recapitulate critical features of GBM, including somatic mutations, antigenic properties and angiogenic activity. More importantly, as we have previously discussed in detail [34,35], PDXs retain the diverse characteristics of individual patient tumors and as such, can effectively recapitulate the intra- and inter-tumor heterogeneity that typifies the clinical disease. By implementing both approaches, we now present a comprehensive preclinical assessment of CDK inhibition, to better inform future therapeutic development in the GBM setting. 

## 2. Results

### 2.1. Seliciclib When Combined with Drozitumab, Reduces Both the Diameter and Viability of Human GBM Neurospheres and Induces Apoptosis In Vitro

**Figure 1:**
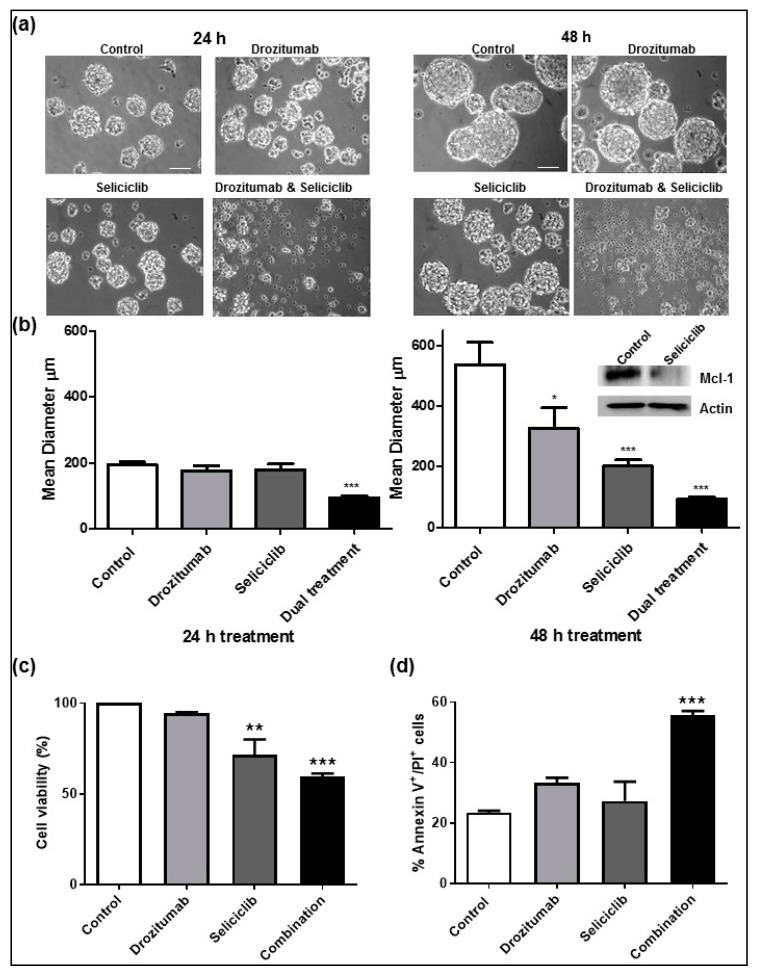
First-generation cyclin-dependent kinase (CDK) inhibitor, seliciclib, in combination with the antibody against human death receptor 5, drozitumab, reduces the diameter and viability of human Glioblastoma (GBM) neurospheres and induces apoptosis in vitro. (**a**,**b**) BT224-luc2 human GBM neurospheres were treated with seliciclib (30 μM) and drozitumab (10 μg/mL) either alone or in combination and the diameter of the human GBM neurospheres was subsequently measured using an Eclipse TE 300 inverted microscope (scale bar = 100 μm). The combination strategy, rather than either treatment alone significantly reduced the diameter of the human GBM neurospheres at 24 h post treatment, and these effects were still evident and statistically significant at 48 h post treatment; *n* = 3 independent experiments and a minimum of 90 human GBM neurospheres were counted for every treatment condition at each time-point. Inset Western Blot analysis of control and seliciclib-treated (30 μM) human GBM neurospheres confirmed that Mcl-1 expression was downregulated upon seliciclib treatment in the neurospheres. Actin was used as a loading control. (**c**) Cell survival was measured following treatment with seliciclib (30 μM) and drozitumab (10 μg/mL) either alone or in combination at 48 h. Data show cell survival relative to control values of 100%. (**d**) Flow cytometry was used to assess the number of PI^+^/AnnexinV^+^ human GBM neurospheres following treatment with seliciclib and/or drozitumab for 48 h. The combination strategy alone induced significant levels of apoptosis within the human GBM neurosphere populations. Data are expressed as mean ± SEM. One-way ANOVA with post-hoc Tukey analysis was used for statistical analysis, whereby, * *p* < 0.05, ** *p* < 0.01, *** *p* < 0.001; *n* = 3 independent experiments performed in triplicate. The uncropped blots and molecular weight markers are shown in supplementary materials.

To investigate if CDK inhibition either alone or in combination with TRAIL represents an important potential future therapeutic strategy for GBM patients, we treated human GBM neurospheres with seliciclib (30 μM) and drozitumab (10 μg/mL), either alone or in combination. These concentrations were selected based on published results [20,33,36]. In addition, survival assays performed using a range of concentrations of both seliciclib and drozitumab (Figure S1), highlighted that at these selected concentrations neither agent was particularly effective as a monotherapy. However, as highlighted in Figure 1a, as early as 24 h after treatment, the combination strategy significantly reduced the diameter of human GBM neurospheres when compared with untreated neurospheres or indeed in comparison to neurospheres treated with either seliciclib or drozitumab alone. The average diameter of control human GBM neurospheres was ~200 μm after 24 h in culture and the average diameter of the drozitumab and seliciclib-treated human GBM neurospheres was ~100 μm after 24 h of treatment. 

This reduction in human GBM neurosphere diameter was maintained over time and remained significant at 48 h (Figure 1b). The average diameter of control untreated human GBM neurospheres was ~500 μm after 48 h in culture and the average diameter of the drozitumab and seliciclib-treated human GBM neurospheres remained approximately ~100 μm after 48 h of treatment. Furthermore, both drozitumab and seliciclib as monotherapies induced a significant reduction in human GBM neurosphere diameter at 48 h post treatment (300 μm and 200 μm, respectively; Figure 1b). Similar to previous results [20], seliciclib successfully targeted the anti-apoptotic Mcl-1 protein in the human GBM neurospheres (Figure 1b inset). Examining cell viability 48 h after treatment, a significant reduction in viability was evident in the seliciclib-treated human GBM neurospheres and the dual-treated human GBM neurospheres (Figure 1c). However, only the combination strategy induced significant levels of apoptosis within the human GBM neurosphere populations (Figure 1d), indicating that the combination treatment of seliciclib plus drozitumab was required to reduce human GBM neurosphere diameter, viability and induce human GBM neurosphere apoptotic death. As we had now observed that the novel drug combination induced significant levels of apoptotic death in both GBM cultured cell lines [20] and in human GBM neurospheres, we next investigated the toxicity and efficacy of the seliciclib and drozitumab combined treatment in an orthotopic GBM PDX model.

### 2.2. In Vivo Toxicity Findings Associated with Seliciclib Plus Drozitumab Combinatorial Regimen

**Figure 2:**
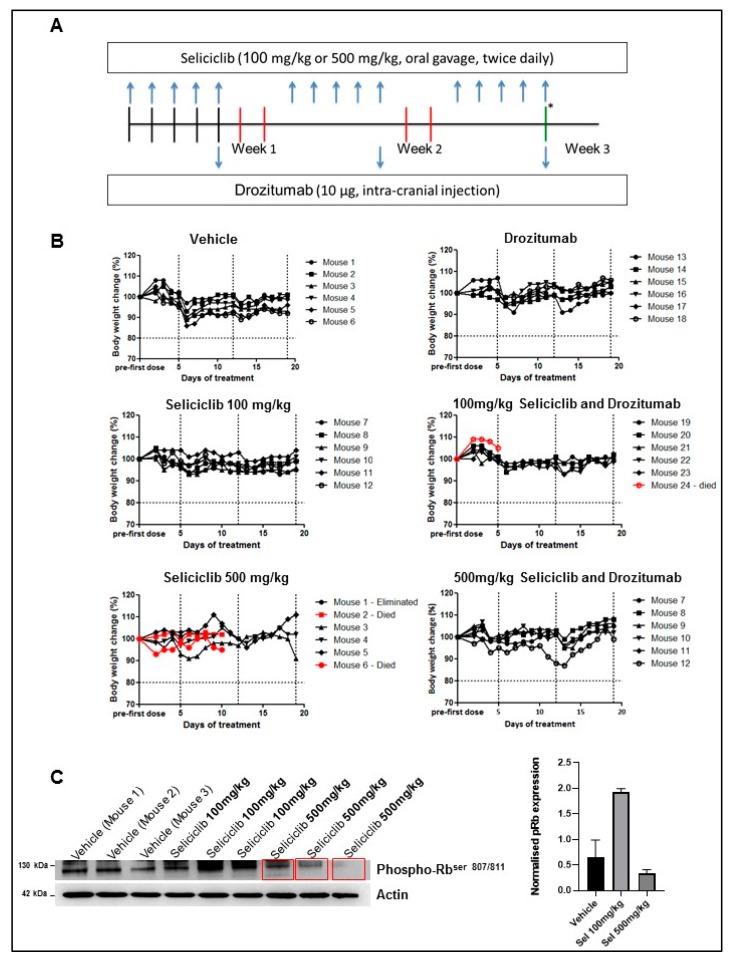
In vivo toxicity findings associated with first-generation CDK inhibitor, seliciclib, in combination with the antibody against human death receptor 5, drozitumab combined treatment. (**A**) Mice were treated as indicated in the toxicity study workflow. Animals were monitored daily and scored for changes in weight loss and behavior as signs of toxic effect. After three weeks, mice were sacrificed by cervical dislocation. (**B**) Body weights of animals that were treated with: (1) vehicles for both routes of drug administration (10% dimethyl sulfoxide (DMSO):5% Tween 20:85% 50 mM hydrochloric acid (HCl)/saline) by oral gavage, twice daily, Monday–Friday, and sterile H_2_O by intracranial injection, once weekly, for three weeks; (2) drozitumab, 10 μg intra-cranially once weekly for three weeks; (3) 100 or 500 mg/kg seliciclib, delivered by oral gavage twice daily, Monday–Friday for three weeks; (4) A combination of seliciclib (100 or 500 mg/kg) plus drozitumab over a three-week period (*n* = 6 per group). (**C**) Western Blot analysis of isolated brain tissue confirmed that the animals that received the higher dose of seliciclib, 500 mg/kg, had a definite and reproducible down-regulation of phospho-Rb. Actin was used as a loading control. The uncropped blots and molecular weight markers are shown in supplementary materials.

To assess the toxicity of this combination strategy in vivo, we held the dose of drozitumab, constant, 10 μg delivered intra-cranially (once weekly) [33], and delivered two escalating doses of seliciclib, 100 and 500 mg/kg, which were administered by oral gavage (twice daily, Monday–Friday) for three-weeks [37] (Figure 2a). 

The effect of treatment on animal weight was monitored (Figure 2b). Three animals that received seliciclib died; two in the 500 mg/kg seliciclib (high concentration)-treated only group after ten doses of seliciclib and one in the combination-treated group after five doses of 100 mg/kg seliciclib (lower concentration). However, none of the animals died in the 500 mg/kg seliciclib (high concentration) and drozitumab combination group. Seliciclib is lipophilic and has been reported to cross the blood brain barrier, albeit concentrations in the brain are approximately 30% lower than those achieved in the plasma [38]. To confirm that seliciclib did reach the brains of the animals in the toxicity study, we examined the expression of a CDK target, phospho-Rb, in the isolated brain tissue of animals and observed a reproducible down-regulation of this target. This downregulation occurred only in the animals that received the higher dose of seliciclib, 500 mg/kg (Figure 2c). Therefore, we selected this higher dose of 500 mg/kg for our future efficacy studies, in combination with 10 μg drozitumab.

### 2.3. In Vivo Efficacy of Seliciclib Plus Drozitumab Combined Treatment in an Orthotopic PDX Model

Animals were orthotopically implanted with human GBM neurospheres (BT224-luc2) and GBM PDX tumor development was monitored using bioluminescence imaging (BLI) (Figure S2a). We confirmed the expression of our drug targets in the human GBM neurospheres (BT224-luc2), both when cultured as neurospheres and in the subsequent tumors that arose upon their orthotopic implantation into the cerebral cortex of severe combined immunodeficiency (SCID) mice. We confirmed that both the neurospheres and the tumors that developed expressed the drozitumab target, DR5 using flow cytometry analysis (Figure S2b) and Western Blot analysis (Figure S2c). We also confirmed that both expressed the seliciclib target Mcl-1, using Western Blot analysis (Figure S2d).

**Figure 3:**
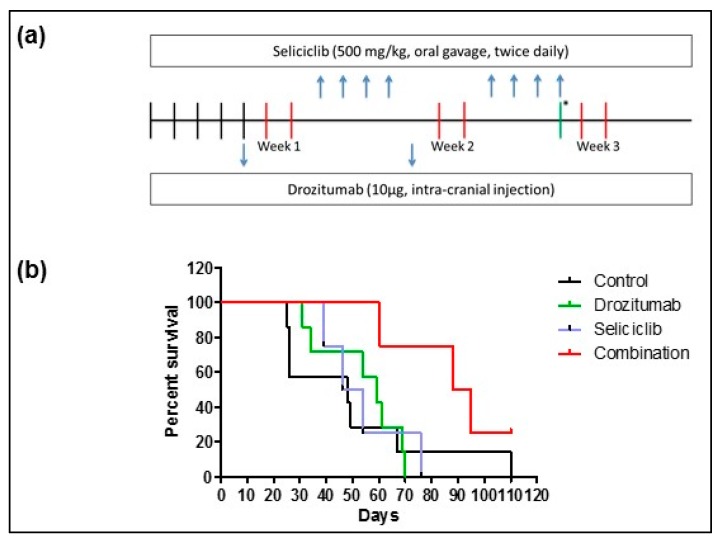
First-generation CDK inhibitor, seliciclib in combination with drozitumab extends survival in GBM orthotopic xenograft mouse model. (**a**) Efficacy study workflow (two-week treatment period); (**b**) Surviving mice were sacrificed at onset of disease symptoms, such as weight loss or behavioral changes, and survival times were analyzed. The extended survival times of the combination-treated group over vehicle and single-treated animals approached significance, log rank *p* = 0.06.

Using BLI to confirm GBM PDX tumor development, 19 weeks post implantation of the human GBM neurospheres (BT224-luc2), tumor-bearing animals were randomized into four groups (*n* = 11/group; Figure 3a). The animals were treated with vehicles (control group) or 10 μg drozitumab, or 500 mg/kg seliciclib or 10 μg drozitumab plus 500 mg/kg seliciclib, over a two week treatment window. Treatment commenced at day 0. Both the seliciclib alone and the seliciclib plus drozitumab combination treatment resulted in toxicity, with five animals from each group succumbing within 48 h of treatment onset. These animals were excluded from subsequent analyses. A trend towards an extended survival time of the combination-treated group, over vehicle and single-treated animals, was observed (*p* = 0.06; Figure 3b). 

**Figure 4:**
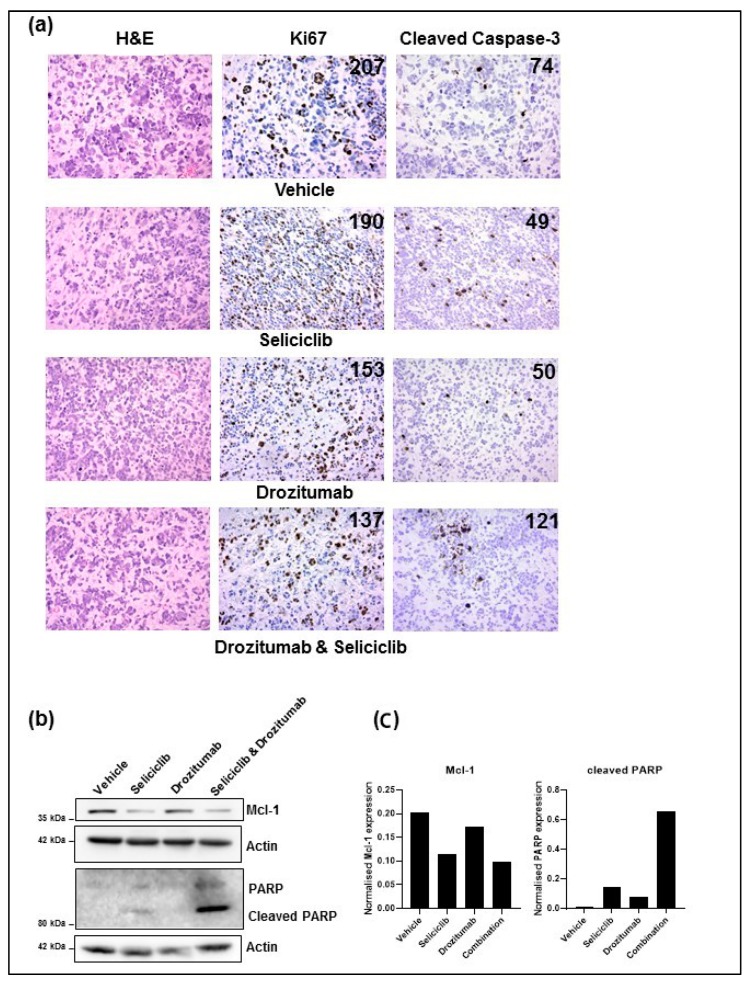
First-generation CDK inhibitor, seliciclib, in combination with the antibody against human death receptor 5, drozitumab, may affect tumor proliferation and induce apoptotic death in vivo. (**a**) Representative photomicrographs (20×) of isolated BT224-luc2 human GBM neurosphere line-derived tumor tissue from mice that died from tumor burden using H&E staining (first column), immunohistochemical staining using Mib-1 antibody for the detection of Ki67 haematoxylin counterstain (middle column) and immunohistochemical staining for cleaved caspase-3 (last column). The images show a slight decrease in the number of proliferating cells (Ki67 positive cells per high power field) between vehicle-treated animals and the three treatment groups (seliciclib, 500 mg/kg; drozitumab, 10 µg; and seliciclib, 500 mg/kg, plus drozitumab, 10 µg). Elevated levels of cleaved caspase-3 were observed in animals treated with seliciclib, 500 mg/kg, and seliciclib, 500 mg/kg plus drozitumab, 10 µg. (**b**) Western Blot analysis of isolated tumor tissue confirmed that Mcl-1 expression was downregulated by both seliciclib alone and in combination with drozitumab. Tubulin was used as a loading control. (**c**) Western Blot analysis of isolated tumor tissue confirmed that the greatest level of cell death was induced by the combination treatment, as evidenced by poly (ADP-ribose) polymerase (PARP) cleavage. Actin was used as a loading control. The uncropped blots and molecular weight markers are shown in supplementary materials.

Post mortem investigations by a veterinary pathologist of animals in the survival analysis that died due to tumor burden (*n* = 1 per treatment group), revealed large tumors in the right hemisphere of the cerebrum in vehicle-treated animals and all treatment groups (Figure 4a; images on left of haematoxylin and eosin (H&E) staining). Ki67 positive cell counts were higher in the control animals compared with treated groups (Figure 4a); elevated levels of cleaved caspase-3 were detected in the seliciclib-treated tumor tissue and in tumors treated with the combination of seliciclib plus drozitumab (Figure 4a). These findings correlated with Western Blot analysis of the isolated tumor tissue, which showed reduced levels of Mcl-1, in both the seliciclib-only treated animals and the animals treated with seliciclib plus drozitumab (Figure 4b). However, tumors from the combination cohort displayed the highest levels of cleaved-PARP, indicating that both seliciclib and drozitumab are required to induce a substantial level of apoptotic death (Figure 4c), in agreement with what was seen in vitro (Figure 1). 

Further post-mortem characterization of brain tissue isolated from an animal that died within 24 h of treatment with the combination of seliciclib and drozitumab revealed that the tumor in this animal extended into the right lateral ventricle and further showed a breach in the blood brain barrier. A similar breach in the blood brain barrier was not evident in the tumor-bearing animals that survived the combination treatment and eventually died from tumor burden in the survival analysis study.

### 2.4. Pharmacodynamics of Second-Generation CDK Inhibitor CYC065, In Vitro and In Vivo

**Figure 5:**
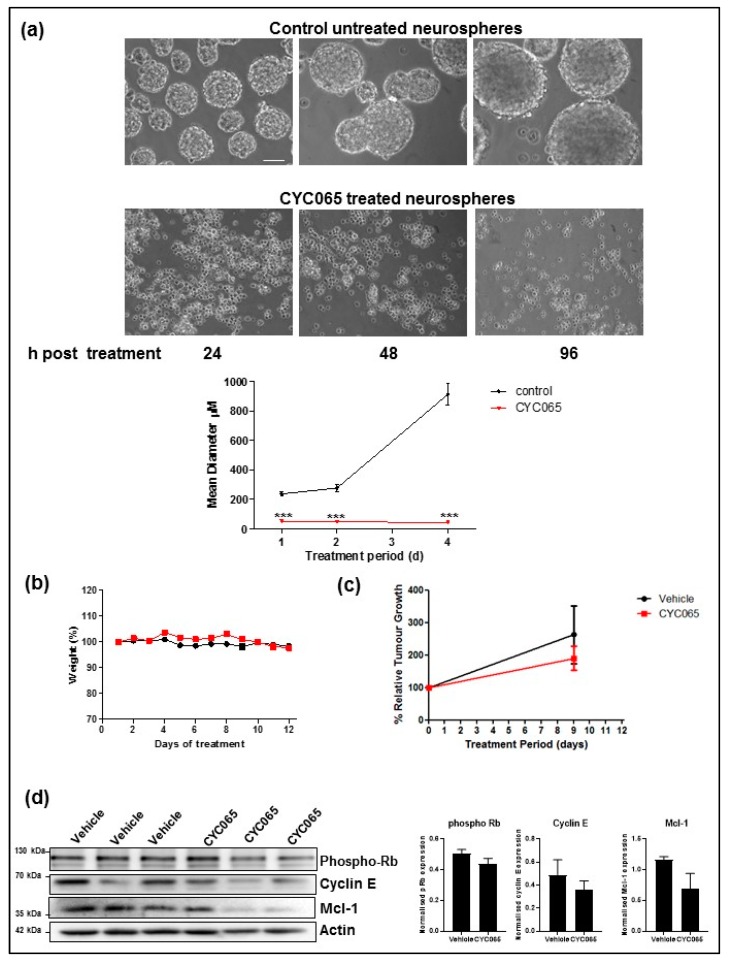
Pharmacodynamics of second-generation CDK inhibitor, CYC065, in vitro and in vivo. (**a**) BT224-luc2 human GBM neurospheres were treated with CYC065 (5 μM) and the diameter of the human GBM neurospheres was subsequently measured using an Eclipse TE 300 inverted microscope (scale bar = 100 μm). CYC065 treatment significantly reduced the diameter of the human GBM neurospheres at 24 h post treatment, and these effects were still evident and statistically significant at 48 h and 96 h post treatment. Two-way ANOVA with Bonferroni post-test was performed for statistical analysis, whereby *** *p* < 0.001; *n* = 3 independent experiments and a minimum of 30 human GBM neurospheres were counted for each treatment condition at each time-point. (**b**) BT224-luc2 human GBM neurosphere line-derived tumor-bearing animals were treated by oral gavage with vehicle (*n* = 4) or one dose of CYC065 (55 mg/kg) daily for nine days (*n* = 5) and were sacrificed at the end of the treatment window. Body weight loss and behavioral changes were used as measures of CYC065-induced toxicity with no observed changes. (**c**) Tumor growth was monitored using bioluminescence imaging. Pre-treatment imaging was carried out at day 0 and end of treatment imaging was carried out at day 9. (**d**) Western Blot analysis of the isolated tumor tissue confirmed that phospho-Rb, Cyclin E and Mcl-1 expression was downregulated by CYC065 treatment. Actin was used as a loading control. The uncropped blots and molecular weight markers are shown in supplementary materials.

As we had administered seliciclib at concentrations exceeding those utilized in the clinic, to ensure efficacy in the brain, we conducted an additional pilot study with a second-generation CDK inhibitor, CYC065. CYC065 is mechanistically similar to seliciclib but with significantly improved potency (40-fold) and metabolic stability [39], facilitating our administration of CYC065 at clinically relevant doses, both in vitro and in vivo. The diameter of 5 μM CYC065-treated human GBM neurospheres grown in vitro was significantly decreased as early as 24 h after treatment (Figure 5a,b). This reduction in diameter was maintained over time, in contrast to control untreated human GBM neurospheres (Figure 5a). GBM tumor-bearing animals were treated by oral gavage with a single dose of CYC065 daily for nine days over a two-week treatment window. Body weight loss was used as a measure of CYC065-induced toxicity; the treatment regimen was well tolerated (Figure 5b). At the end of the treatment window, there was a trend towards a reduction in tumor volume (Figure 5c). Animals were sacrificed and tumors isolated. Western Blot analysis of CYC065 targets, cyclin E and Mcl-1, in excised tumors indicated that CYC065 delivered by oral gavage successfully crossed the BBB, as there was a reduction in these proteins in the CYC065-treated tumors but not the vehicle-treated tumors (Figure 5d).

## 3. Discussion

By implementing advanced preclinical models, we have demonstrated in this study the potential of CDK inhibition, both alone and in combination with death receptor ligand, as a potential treatment option for GBM. Due to seliciclib’s low brain penetrance, the dosing regimen required to observe seliciclib-targeted responses in the brain, whether delivered as a single agent or in combination with drozitumab, exceeded clinical levels and lethal side-effects were observed. However, the in vitro effects of the combination treatment were encouraging: reduction in both human GBM neurosphere diameter and viability, in addition to the induction of human GBM neurosphere apoptotic death. Increased survival times in the mice that tolerated the treatment regime were also observed. Therefore, future efforts should focus on different dosing routes for first-generation inhibitors or substituting with second-generation inhibitors such as CYC065, that have better dose potency and improved therapeutic index.

CDKs are a family of enzymes first discovered as regulators of the cell cycle, but are now understood to also have pivotal functions in the regulation of transcription, DNA repair and metastatic spread [40]. As a result, there has been tremendous interest in the clinical applicability of CDK inhibitors as anti-cancer agents [40,41]. The first generation of CDK inhibitors developed were relatively nonspecific and were often classified as “pan CDK” Inhibitors [40]. Of the first-generation inhibitors, flavopiridol is the most extensively investigated [40]. Flavopiridol has demonstrated promising results in combination with TMZ in GBM cell lines and xenografts [42], but has not undergone clinical testing in patients. We and others have demonstrated that seliciclib, another first-generation CDK inhibitor and one of the first to be evaluated in the clinic, can induce significant levels of apoptosis in GBM cell lines upon co-treatment with the death receptor ligand, TRAIL [20,43]. The current work provides further evidence of the utility of this combination to induce apoptosis in human GBM neurospheres in vitro and in a PDX model in vivo. 

Seliciclib has been associated with diverse cellular effects, including cell cycle inhibition, transcriptional suppression, and apoptosis induction [40]. In the current study, we have demonstrated apoptotic cell death within human GBM neurospheres and suppression of Mcl-1 expression in vitro and in vivo, upon administration of seliciclib alone. With the potential to target multiple critical cellular pathways, it is important that such CDK inhibitors undergo rigorous toxicity assessment in the pre-clinical stages. Herein, treatment with seliciclib alone, or in combination with drozitumab, was well tolerated by the animals in the toxicity studies. Three deaths were observed however, two in the 500 mg/kg seliciclib-treated only group after ten doses of seliciclib and one in the combination-treated group after five doses of 100 mg/kg seliciclib. None of the animals died in the 500 mg/kg seliciclib and drozitumab combination group. However, during PDX efficacy studies, significant toxicity was observed in vivo following both single agent administration of seliciclib and when the drug was administered in combination with drozitumab. Detailed, post mortem investigations by a Veterinary Pathologist did not reveal the specific cause for the lethal toxicity observed in the PDX bearing animals. Nevertheless, we hypothesize that brain tumor burden along with the related changes observed in the blood brain barrier, may have resulted in increased drug delivery to the brains of the PDX bearing animals, which in turn account for the differences observed in survival between the mice from the efficacy study and the mice from the toxicity study. These results highlight the critical need to perform rigorous toxicity studies under the most appropriate conditions, particularly implementing PDX models.

Second-generation CDK inhibitors have recently been developed that inhibit CDKs with better dose potency. We thus performed additional studies with the second-generation CDK inhibitor, CYC065 that is more potent against CDK-2, -5 and -9 than seliciclib. Administration of CYC065 to tumor bearing animals over a 2-week treatment window was well tolerated with no deaths observed. 

CYC065 causes proportionally greater CDK9 inhibition than seliciclib, leading to prolonged down-regulation of apoptotic proteins, such as Mcl-1. We observed downregulation of Mcl-1 in the brains of tumor-bearing animals after treatment with 55 mg/kg CYC065 over a 9-day period (compared with a 14-day treatment window with 500 mg/kg seliciclib). Such downregulation of Mcl-1 was sufficient to trigger apoptosis in CYC065-only treated human GBM neurospheres (Figure S3). We also observed possible evidence of cell cycle targeting via CDK2, as cyclin E was downregulated in the brains of CYC065-treated tumor-bearing animals. The potential of CYC065 to target CDK5 is particularly encouraging in this disease paradigm. CDK5 is highly expressed in GBM [44], possibly due to its location on chromosome 7, which is one of the most frequent sites of copy number gains in primary (isocitrate dehydrogenase [IDH] wild-type) GBM [45]. Aberrant CDK5 activity plays a critical role in the growth and propagation of multiple forms of cancers [44,46]. Moreover, it has been shown that CDK5 promotes the migration and invasion of glioma cells [44,47]. More recent studies suggest that targeting CDK5 signaling to treat GBM could potentially eliminate CDK5-addicted glioma stem cells and reduce the likelihood of recurrence [48]. Finally, CYC065 is orally bioavailable and lipophilic in nature. Thus, if its therapeutic potential is realized it would have the propensity to cross the BBB in humans. We have also provided evidence in this study to suggest that CYC065 is able to cross the BBB in mice-phosphor-Rb, Cyclin E and Mcl-1 downregulation within the tumor tissue of CYC065-treated animals. 

There is increasing evidence that rational combination strategies with standard of care or other molecularly targeted agents are particularly important to exploit the therapeutic potential of CDK inhibitors. TRAIL is an anti-cancer therapy that has been gaining momentum in recent years [16,49,50,51,52]. Using extrinsic agents like TRAIL has numerous advantages: Firstly, TRAIL can trigger apoptosis independently of p53, which is commonly mutated in GBM patients [53], contributing, in part, to TMZ resistance [54]; and secondly, TRAIL can kill cancer cells without conferring significant toxicity to normal cells [51]. To target the TRAIL signaling pathway, we selected a monoclonal antibody specifically directed against human TRAIL-R2 (drozitumab) as GBM cell lines predominately express TRAIL-R2 [33]. Drozitumab has previously been shown to be effective in GBM cells [33] but has not been investigated in human GBM neurospheres or indeed an orthotopic PDX model.

We observed significant levels of apoptosis in both the in vitro and in vivo settings upon administration of drozitumab in combination with seliciclib. Somewhat encouragingly, the extended survival times of animals, that tolerated the combination treatment in vivo, approached significance when compared with vehicle cohort. A limitation of this current study was the lack of funding to study CYC065 in combination with drozitumab in vivo. Future studies are planned to examine this combination. Mcl-1 overexpression could be a potential pharmacodynamic marker for this treatment strategy [55]. 

## 4. Materials and Methods

### 4.1. Materials

Seliciclib, (R-Roscovitine, CYC202) and CYC065 were kindly gifted by Cyclacel Ltd. (Dundee, UK). Drozitumab, (PRO95780, Apomab) was kindly gifted by Genentech Inc. (San Francisco, CA, USA). 

### 4.2. Human GBM Neurosphere Culture 

Human GBM neurosphere line, BT224-luc2 was donated by Dr Keith L Ligon of the Dana-Farber Cancer Institute, Boston, Massachusetts, USA; BT224-luc2 was derived from a patient at Brigham and Women’s Hospital undergoing surgery according to Institutional Review Board (IRB)-approved protocols, as previously described [56,57]. Luciferase expression of human GBM neurosphere lines was performed using retrovirus encoding a fusion of luciferase and neomyocin phosphotransferase (pMMP-LucNeo), as previously described [58]. 

Human GBM neurospheres were cultured in Complete Human NeuroCult^TM^ NS-A Proliferation Medium (Stemcell Technologies, Cambridge, UK) containing Human NeuroCult^TM^ NS-A Proliferation Supplement (Stemcell Technologies, Cambridge, UK), rh-EGF (20 ng/mL; Miltenyi Biotec Ltd., Surrey, UK), rh-bFGF (10 ng/mL; Miltenyi Biotec Ltd., Surrey, UK), and 0.2% Heparin (2 μg/mL; Stemcell Technologies, Cambridge, UK). Human GBM neurospheres were grown in Corning Ultra-Low Attachment T25 cm^2^ or T75 cm^2^ Flasks (Sigma-Aldrich Ireland Ltd, Dublin, Ireland) and maintained at 37 °C in a 5% CO_2_ humidified incubator. Neurospheres were mechanically dissociated when they reached approximately 100–150 μm in diameter and passaged no more than twelve times.

### 4.3. Light Microscopy 

BT224-luc2 human GBM neurospheres were treated with seliciclib (30 μM) and/or drozitumab (10 μg/mL) or CYC065 (5 μM), as indicated for either 24 h or 48 h or 96 h. The effect of treatment on neurosphere formation and viability was monitored using an Eclipse TE 300 inverted microscope (Nikon) with a 20× objective. Neurosphere diameter was quantified from 16-bit grayscale images using a script (Data S1) for Fiji [59].

### 4.4. Cell Viability Assay

BT224-luc2 human GBM neurospheres were plated in 96-well plates (3000 cells/well) and treated with increasing concentrations of drozitumab, seliciclib or their combination. Following 48 h of treatment, WST-1 reagent (Sigma-Aldrich) was added in 1:10 final dilution, according to the manufacturer’s instructions. WST-1 salt is cleaved to a soluble formazan dye by a NAD(P)H-dependent reaction in viable cells. Plates were incubated for 3 h in a humidified incubator at 37 °C and 5% CO_2_ and the absorbance of each sample was measured at 450 and 620 nm using a microplate reader (GENios, Tecan, Reading, UK). The absorbance was proportional to the number of viable cells and expressed relative to control treated samples.

### 4.5. Flow Cytometry 

The basal surface expression of DR5 on BT224-luc2 human GBM neurospheres was assessed using a BD LSR II flow cytometer (BD Biosciences, Berkshire, UK). Briefly, 1 × 10^5^ neurospheres were transferred to FACs tubes and pelleted by centrifugation at 1200 rpm for 5 min at 4 °C. Following incubation with a blocking buffer (0.5% BSA) on ice for 20 min, neurospheres were incubated with a mouse monoclonal antibody against DR5 (Cat# ALX-804-914-0100, Enzo Life Sciences, Exeter, UK; 1:100 dilution) for 30 min, pelleted and washed × 3 with phosphate buffered saline (PBS), and then incubated with a secondary anti-mouse fluorescein isothiocyanate (FITC)-conjugated antibody (Life Technologies, Invitrogen, Paisley, Scotland; 1:200 dilution) for 30 min in the dark. Controls were stained with secondary antibody only. FITC was excited at 488 nm and fluorescence emission was collected in the FL1 channel through a 520 nm band-pass filter. A total of 1 × 10^4^ gated cells were acquired. The relative expression of DR5 was determined by comparison of specific staining intensities compared to individual cell line negative controls. For cell death measurements, flow cytometry was used to assess the number of PI/AnnexinV^+^ BT224-luc2 human GBM neurospheres following treatment with seliciclib (30 μM) and/or drozitumab (10 μg/mL) or CYC065 (5 μM) for 48 h. Briefly, 1 × 10^5^ neurospheres were transferred to FACs tubes and pelleted by centrifugation at 1200 rpm for 5 min at 4 °C. Following appropriate treatment conditions, neurospheres were pelleted and incubated in 100 μL of binding buffer (10 nM 4-(2-hydroxyethyl)-1-piperazineethanesulfonic acid (HEPES), 135 nM NaCl, 5 mM CaCl2) containing AnnexinV-FITC conjugate (5 μL/mL) (BioVision, Moutain View, CA, USA) and propidium iodide (PI) for 10 min on ice in the dark. FITC was excited at 488 nm and fluorescence emission was collected in the FL1 channel through a 520 nm band-pass filter. PI was excited at 561 nm and fluorescence emission was collected through a 605/40 nm band-pass filter and a 570 nm long pass filter. A total of 1 × 10^4^ gated cells were acquired.

### 4.6. Toxicity Assessment of Seliciclib Plus Drozitumab Combined Treatment in Rodent Models 

All animal studies were licensed by the Health Product Regulatory Authority (HPRA), Dublin, Ireland (project authorization number AE19127/PO28). All protocols were also approved by the Royal College of Surgeons in Ireland (RCSI) Animal Research Ethics Committee (AREC; project reference number REC1068bbbbb). To monitor the potential toxic effect of seliciclib plus drozitumab combination treatment, a dose escalation study was performed in non-tumor-bearing SCID mice. Mice were randomised into six groups, with *n* = 6 per cohort: Vehicle, seliciclib 100 mg/kg, seliciclib 500 mg/kg, drozitumab 10 μg, seliciclib 100 mg/kg + drozitumab 10 μg and seliciclib 500 mg/kg + drozitumab 10 μg. Seliciclib (100 or 500 mg/kg), dissolved in 10% DMSO: 5% Tween 20:85% 50mM HCl/saline, was administered by oral gavage (twice daily, Monday–Friday, for three weeks). Drozitumab (10 μg), dissolved in sterile H_2_O, was administered by intracranial injection (once weekly, for three weeks) (see below for protocol). Vehicle-treated mice received vehicles for both routes of drug administration (10% DMSO:5% Tween 20:85% 50 mM HCl/saline by oral gavage, twice daily, Monday–Friday, for three weeks and sterile H_2_O by intracranial injection, once weekly, for three weeks. Mice were monitored daily and scored for changes in weight loss and behavior as signs of toxicity. After three weeks, mice were sacrificed by cervical dislocation. 

### 4.7. Assessment of Seliciclib Plus Drozitumab Combinatorial Regimen in a PDX Model 

All animal studies were licensed by the Health Product Regulatory Authority (HPRA), Dublin, Ireland (project authorization number AE19127/PO28). All protocols were approved by the Royal College of Surgeons in Ireland (RCSI) Animal Research Ethics Committee (AREC project reference number REC1068bbbbb and REC1173bb). Female SCID mice (4–6 weeks old) (CB17/lcr-Prkdcscid/lcrlcoCrl) were purchased from Charles River Laboratories (Canterbury, UK) and Harlan Laboratories UK Ltd (Bicester, UK) and housed in an isolated facility within a specific pathogen-free (SPF) environment. Orthotopic implantation of PDX neurospheres was carried out as previously described [60,61]. Briefly, animals (*n* = 44) were anaesthetized with isoflurane (4% for induction, 1.5% for maintenance) in 100% oxygen (1.5 L/min), and a burr hole was prepared 2 mm right of bregma using a microdrill. BT224-luc2 human GBM neurospheres (1 × 10^5^) were then slowly injected into the cerebral cortex using a Hamilton syringe (Hamilton, Bonaduz, Switzerland) at a depth of 2 mm. Incisions were closed with Ethilon 3–0 sutures (Ethicon, NJ, USA) and mice were returned to their cages for recovery. Treatment commenced at week 19 with presence of tumor confirmed using bioluminescence imaging (BLI). Mice were randomized into four groups (*n* = 11 per group): Vehicle, seliciclib 500 mg/kg, drozitumab (10 μg), and seliciclib 500 mg/kg + drozitumab (10 μg). Seliciclib and drozitumab were administered as described above, with drozitumab injected intracranially into the same site as BT224-luc2 neurospheres. For survival analysis, mice were sacrificed at onset of disease symptoms, such as weight loss or behavioral changes.

### 4.8. BLI

Mice received an intraperitoneal (i.p.) injection of D-luciferin (150 mg/kg) and images were recorded 10 min later using the IVIS Spectrum scanner and analyzed using Live Imaging 3.2 software (Caliper Life Sciences, Waltham, MA, USA); animals were shaved prior to image acquisition in order to improve the bioluminescent signal, and imaging was carried while mice were anaesthetized with isoflurane (4% for induction, 1.5% for maintenance) in 100% oxygen (1.5 L/min). BLI commenced four weeks after neurosphere implantation and was performed thereafter on a weekly basis to monitor tumor growth.

### 4.9. Pharmacodynamic Analysis of Second-Generation CDK Inhibitor CYC065, in a GBM Orthotopic PDX Model 

The study was performed under the same HPRA project authorization number (AE19127/PO28) and AREC project reference numbers (REC1068bbbbb and REC1173bb) as described above. Female SCID mice (4–6 weeks old) (CB17/lcr-Prkdcscid/lcrlcoCrl) were purchased from Charles River Laboratories (Canterbury, UK) and Harlan Laboratories UK Ltd (Bicester, UK) and housed in an isolated facility within a SPF environment. Orthotopic implantation of neurospheres was carried out as described above. Mice (*n* = 9) were randomized into two groups, with *n* = 4 for vehicle group and *n* = 5 for CYC065 55 mg/kg group [62]. CYC065 (55 mg/kg), dissolved in sterile water, was administered by oral gavage for 2 weeks. Vehicle group mice acted as a control and received sterile water by oral gavage for 2 weeks. Mice were monitored daily and scored for changes in weight loss and behavior as signs of toxic effect. Mice were sacrificed by cervical dislocation. 

### 4.10. Western Blot Analysis 

BT224-luc2 human GBM neurospheres and mouse tissues were homogenized in lysis buffer containing 0.5 mmol/L Tris-HCl (pH 6.8), 2% SDS (*w*/*v*), 10% glycerine (*w*/*v*), and protease and phosphatase inhibitor cocktails (Sigma-Aldrich). After determining the protein concentration of samples using a BCA protein assay (Pierce, Rockford, IL, USA), 20 μg samples were boiled in gel-loading buffer and separated on 10–15% SDS-PAGE gels. Proteins were transferred to nitrocellulose membranes using the iBlot gel transfer device (Life Technologies, Invitrogen, Paisley, Scotland). The membranes were blocked in either 5% non-fat milk or 5% BSA (for phosphorylated proteins) in TBST for 1 h at room temperature prior to being incubated with primary antibodies overnight at 4 °C. The following primary antibodies were used: Mcl-1 (Cat# SC-819, Santa Cruz Biotechnology Inc., Dallas, TX, USA); DR5 (Cat# ALX-804-914-0100, Enzo Life Sciences); phospho-Rbser 807/811 protein (Cat# ABC132, Sigma-Aldrich); PARP and cleaved PARP (Cat# 9542, Cell Signaling Technology, Danvers, MA, USA), Cyclin E (Cat# SC-247); β-actin (Cat# A5441, Sigma-Aldrich); and α-tubulin (Cat# T6199, Sigma-Aldrich). Membranes were then washed three times with tris-buffered saline, 0.1% Tween 20 detergent (TBST) for 5 min prior to being incubated with peroxidase-conjugated secondary antibodies (anti-mouse or anti-rabbit; MilliporeSigma, Danvers, MA, USA) for 1 h at room temperature. Protein bands were visualized using the Immobilin western chemiluminescence HRP substrate (Millipore Sigma) and images were captured using a LAS-4000 imager equipped with a cooled 12 bit digital CCD camera (Fujifilm UK Ltd, Bedfordshire, UK). To guarantee accurate quantifications, special care was taken not to over-expose the protein bands. Densitometry was carried out on 12-bit raw images using ImageJ 1.4.10 software (National Institute of Heath, Bethesda, MD, USA; http://rsb.info.nih.gov/ij).

### 4.11. Histological Assessment and Immunohistochemistry 

Mouse brains were excised, rinsed twice in D-PBS and fixed in 4% formaldehyde for 48 h. Coronal sections (3 mm) of the brain tumors were placed into cassettes and processed to paraffin embedded tissues using standard histopathological techniques. Tissues from each paraffin block were cut at 4 μm. Sections were deparaffinized and re-hydrated according to standard histological procedures. Routine hematoxylin and eosin (H&E) staining was performed to facilitate histological evaluation by a veterinary pathologist. Immunohistochemistry was performed on the Leica Bond III automated immunostaining platform. This system includes a peroxidase block and protein blocking agents. Heat induced epitope retrieval using Leica ER2 solution (pH9, Leica biosystems, Wetzlar, Germany) for 20 min was performed. This was followed by primary antibody incubation, either mouse monoclonal anti-Ki67 assessing cell proliferation, MIB1 (Dako M7240, Gostrup, Denmark; dilution 1 in 75 for 20 min) or rabbit polyclonal anti-cleaved caspase 3, assessing cell apoptosis, ASP175 (Cell Signaling Technology #9661, New England Biolabs; dilution of 1 in 300 for 30 min). Primary antibodies were visualised using Leica Bond Polymer Refine detection (Leica Biosystems Inc., Buffalo Grove, IL, USA) with a DAB chromogen according to the manufacturer’s instructions. Sections were counterstained with hematoxylin (Sigma-Aldrich) to visualise nuclei. Negative controls were performed by omitting the first step with primary antibody and yielded negative results. Intestine and tonsils were used as positive controls. The average number of cells expressing Ki67 or cCasp-3 per high power field (400×, 0.55 mm diameter) was calculated by counting the number of cells positively labeled by the relevant antibody in three different fields manually using light microscopy.

### 4.12. Statistical Analyses 

Statistical analysis of all data was carried out on GraphPad InStat software. Statistical analysis of the in vitro data was performed using one-way ANOVA followed by Tukey’s post hoc test or two-way ANOVA where appropriate. Statistical analysis of the in vivo data was done by 2-way ANOVA for BLI and by a log-rank test for survival studies. When the *p* value was < 0.05, groups were considered to be significantly different. 

## 5. Conclusions

Our data support the potential future development of a CDK inhibition strategy either alone or in combination with death receptor ligation as a potential treatment approach for GBM. Future studies should focus on alternative dosing routes for first-generation inhibitors due to toxicities associated with systemic exposure to high concentrations of these compounds. Second-generation inhibitors could also be utilized as suitable alternatives. This study highlights the continued need for robust pre-clinical assessment of promising combinatorial treatment approaches using clinically relevant GBM models.

## Supplementary Materials

The following are available online at https://www.mdpi.com/2072-6694/11/12/2005/s1, Figure S1: BT224-luc2 human GBM neurospheres are resistant to treatment with drozitumab alone and moderately sensitive to treatment with seliciclib alone; Figure S2: BT224-luc2 human GBM neurospheres give rise to GBM PDX tumours upon their orthotopic implantation and express DR5 and Mcl-1 in vitro and in vivo; Figure S3: Second-generation CDK inhibitor, CYC065, induces moderate levels of apoptosis in in vitro GBM neurosphere model. Data S1: Script to measure human GBM neurosphere diameter.
